# Both absolute and relative quantification of urinary mRNA are useful for non-invasive diagnosis of acute kidney allograft rejection

**DOI:** 10.1371/journal.pone.0180045

**Published:** 2017-06-27

**Authors:** Jung-Woo Seo, Haena Moon, Se-Yun Kim, Ju-Young Moon, Kyung Hwan Jeong, Yu-Ho Lee, Yang-Gyun Kim, Tae-Won Lee, Chun-Gyoo Ihm, Chan-Duck Kim, Byung Ha Chung, Yeong Hoon Kim, Sang Ho Lee

**Affiliations:** 1 Department of Internal Medicine, Division of Nephrology, College of Medicine, Kyung Hee University, Seoul, South Korea; 2 Department of Biomedical Science, Graduate School, Kyung Hee University, Seoul, South Korea; 3 Department of Internal Medicine, Division of Nephrology, Kyung-pook National University School of Medicine, Daegu, South Korea; 4 Department of Internal Medicine, Division of Nephrology, Seoul St. Mary’s Hospital, College of Medicine, The Catholic University of Korea, Seoul, South Korea; 5 Department of Internal Medicine, Division of Nephrology, Busan Paik Hospital, College of Medicine, Inje University, Busan, South Korea; University Jean MONNET of SAINT-ETIENNE, UNITED STATES

## Abstract

Urinary mRNA analysis with three-gene set (18S rRNA, CD3ε, and IP-10) has been suggested as a non-invasive biomarker of acute rejection (AR) in kidney transplant recipients using quantitative real-time PCR (qPCR). Application of droplet digital PCR (ddPCR), which has been suggested to provide higher sensitivity, accuracy, and absolute quantification without standard curves, could be a useful method for the quantifying low concentration of urinary mRNA. We investigated the urinary expression of these three genes in Korean patients with kidney transplantation and also evaluated the usefulness of ddPCR. 90 urine samples were collected at time of allograft biopsy in kidney recipients (n = 67) and from patients with stable renal function more than 10 years (n = 23). Absolute quantification with both PCR system showed significant higher mRNA levels of CD3ε and IP-10 in AR patients compared with stable transplants (STA), but there was no difference in 18S rRNA expression across the patient groups. To evaluate discrimination between AR and STA, ROC curve analyses of CTOT-4 formula yielded area under the curve values of 0.72 (95% CI 0.60–0.83) and 0.77 (95% CI 0.66–0.88) for qPCR and ddPCR, respectively. However, 18S normalization of absolute quantification and relative quantification with 18S showed better discrimination of AR from STA than those of the absolute method. Our data indicate that ddPCR system without standard curve would be useful to determine the absolute quantification of urinary mRNA from kidney transplant recipients. However, comparative method also could be useful and convenient in both qPCR and ddPCR analysis.

## Introduction

Kidney-derived cells exist in the urine of both healthy individual and kidney transplant patient, and these cells contain various molecules associated with ongoing kidney injury or allograft status. Development of noninvasive biomarkers within human urine would therefore be useful for kidney disease monitoring. In 2001, Suthanthiran *et al*. [1] first reported that mRNA levels of granzyme B and perforin were increased in the urinary cells of patients diagnosed with AR by biopsy, and it was suggested that measurement of these mRNA levels in urine could be a potential noninvasive AR diagnostic tool. Recently, the multicenter Clinical Trials in Organ Transplantation-04 (CTOT-4) reported that the three-gene signature of CD3ε, IP-10, and 18S rRNA in urinary cells of kidney recipients discriminated between patients with AR and those with no rejection using the absolute PCR quantification method [2].

Analysis of mRNAs from clinical urine samples is still a challenging due to several reasons such as low amounts, storage conditions, RNA quality, PCR amplification efficiency, and so on [3, 4]. Normalization is necessary to correct expression data for these variations between clinical samples. Usually, if the expression of the selected housekeeping gene is stable and ubiquitous, normalization by the housekeeping gene is an easy and widely used method [5]. However, the selection of the best optimal gene for normalization is still the issue of debate due to unstable expression according to clinical sample conditions. Normalized urinary mRNAs by the total amount of RNA developed by Suthanthiran et al. [2] discriminated patients with AR from those with no AR, but normalization of target mRNA using 18S rRNA or other housekeeping genes is still an issue of debate [4–9]. Another method of analyzing real-time PCR data is the relative quantification known as the 2^-*ΔΔC*т^ method, which is more convenient and widely using method in biologic experiments [10, 11].

Diagnostic tool to monitor kidney allograft rejection or dysfunction should be fast, easy, and simple for clinical trials. Quantitative real-time PCR (qPCR) system has been favorably and conveniently used by many researchers, and absolute and relative quantification are commonly used to analyze data [10, 12]. In quantitative real-time PCR system, absolute copy numbers of genes are calculated with standard curves. Although the qPCR system is well established and robust, there are some limitations, such as low sensitivity and efficiency when detecting low concentration of target genes. In addition, each standard curve of targets is required for the absolute quantification. The droplet digital PCR (ddPCR, BioRad QX200) system advanced in general qPCR provides several advantages, including enhanced sensitivity to partial inhibition of target gene amplification, robustness in the presence of PCR efficiency variations, and absolute quantification of the target without a standard curve [13].

In this validation study for the CTOT-4 formula of urinary mRNAs in Korean kidney transplant recipients, who have different genetic and demographic features from American kidney transplant recipients, we slightly modified the PCR method used by Suthanthiran *et al*. [2] for considering easily degradable nature of mRNA in urine samples, possible errors in the measurement of total amount of RNA and pre-amplification step which was attempt to screen more numbers of target mRNAs. In the modified PCR method, we used every standard curve of three genes for absolute quantification, and did not perform pre-amplification step. Furthermore, we evaluated whether ddPCR system to absolutely quantify three genes without standard curve is promising and whether relative quantification with the 2^-*ΔΔC*т^ method is also useful to monitor kidney allograft rejection in real-time PCR analysis.

## Materials and methods

### Patients and sample preparation

All of the studied patients were chosen from ARTKT-1 (assessment of immunologic risk and tolerance in kidney transplantation) study, which was a cross sectional sample collection study for renal allograft recipients who underwent graft biopsy or who have long-term graft survival (LGS) with stable kidney function (eGFR ≥ 50 ml/min/1.73m^2^) over 10 years at five different transplantation centers (Kyung Hee University Hospital at Gangdong, Kyung Hee University Hospital, Kyungpook National University Hospital, Samsung Medical Center and St. Mary’s Hospital of Catholic University of Korea) from August 2013 to July 2015.

Among the samples which were collected during first year of the study, a total of 67 samples from the patients of category 1 (n = 21), 2 (n = 15) and 4 (n = 31) on graft biopsy with Banff classification assessed by a single pathologist and the remaining 23 samples from the patients with LGS were selected for this study. Samples from Banff category 1 and LGS were grouped as stable graft function (STA). We used the Modification of Diet in Renal Disease (MDRD) equation to estimate the GFR.

At the time of transplantation, none of the transplant donors were from a vulnerable population and all donors or next of kin provided written informed consent that was freely given. All studied patients provided written informed consent prior to participation in the study. This study was approved by the local institutional review board (#2012–030, Institutional Review Board of Kyung Hee University Hospital) and registered in Clinical Research Information Service (KCT0001010).

Urine samples (approximately 50 ml) from the KTPs in each center were collected at the time of biopsy using an identical protocol. The pellets transferred into RNA*later* (Invitrogen, Carlsbad, CA) were stored at -80°C until later use. Total RNA from the urinary cell pellets was extracted using the PureLink^™^ RNA Mini Kit (Invitrogen) according to the manufacturer’s recommendations. The quantity (absorbance at 260nm) and purity (ratio of the absorbance at 260nm and 280nm) of the RNA were measured using the NanoDrop^®^ ND-2000 UV spectrophotometer (Thermo Scientific). The median (25^th^ and 75^th^ percentile) of the quantity (ug) of total RNA amount isolated from 90 samples was 0.330 (0.154–0.649), and the median (25^th^ and 75^th^ percentile) of the purity of total RNA was 1.93 (1.81–2.05).

### Real-time PCR (qPCR) analysis

RNA was reverse-transcribed into cDNA using M-MLV Reverse Transcriptase system (200 U/μl; Mbiotech, Inc., Seoul, Korea) in a 25-ul total volume. Gene-specific oligonucleotide primers and TaqMan probes were used for the measurement of CD3ε, IP-10, and 18S rRNA levels in the two PCR systems. TGF-β1 (assay ID; Hs00998133_m1, Applied Biosystems, Foster City, CA, USA) and 18S rRNA were used as QC parameters. Urine samples with a qPCR-determined 18S rRNA copy number greater than or equal to 5x10^5^ copies per microgram of total RNA and a TGF-β1 mRNA copy number greater than or equal to 100 copies per microgram of total RNA passed quality control and were used in the analysis. The commercially Universal Human Reference RNA (Agilent Technologies, Santa Clara, CA, USA) and 18S rRNA were used for the 2^-*ΔΔC*т^ method [14].

Absolute levels of the mRNAs were calculated using the standard curve method. Standard DNA fragments of CD3ε, IP-10, and 18S rRNA were synthesized by Integrated DNA Technologies (IDT, Coralville, IA, USA). Each gene fragment stock solution of 1 ng/μl was serially diluted from 1x10^-1^ to a working solution of 1x10^-8^ ng/μl for each standard curve, and the serially diluted solution was amplified with each gene-specific primer pair and TaqMan probe using an ABI StepOnePlus real-time PCR system (Applied Biosystems). The threshold cycle (C_T_) value of each target was converted to a concentration using the appropriate standard curve.

Gene expression was performed using real-time PCR with the standard TaqMan protocol (10 min at 95°C, 40 cycles of 15 sec at 95°C and 60 sec at 60°C) in a 96-well microplate with each reaction mixture containing 1 μl of cDNA, 10 μl of TaqMan Universal PCR Master Mix, No AmpErase UNG, 0.9 μM primers, and 0.25 μM probes in 20 μl. Quantities were calculated from a standard curve, and the number of copies was converted using the molecular weight of DNA [15].

### Droplet digital PCR (ddPCR) analysis

The same assay was performed using the QX200^™^ Droplet Digital PCR System (Bio-Rad, Hercules, CA, USA) with the 20-μl reaction mixtures containing 0.9 μM primers, 0.25 μM probes, 1x ddPCR Supermix for Probes (Bio-Rad), 1 μl of cDNA, and RNase- and DNase-free water. In brief, each reaction mixture was mixed with 70 μl of Droplet Generation Oil (Bio-Rad) in a disposable cartridge, partitioned into approximately 20,000 nanoliter-sized droplets in the QX200 Droplet Generator (Bio-Rad), and then transferred into 96-well plates (Eppendorf) and sealed. The Bio-Rad T100 thermal cycler was used for PCR amplification with the following cycling conditions: 10 min at 95°C; 40 cycles of 30 sec at 94°C and 60 sec at 57°C; and 1 cycle of 10 min at 98°C with a 2°C/s ramp rate. At the end of PCR amplification protocol, the droplets were read individually with the QX200 Droplet Reader (Bio-Rad) and quantified with QuantaSoft droplet reader software (Bio-Rad). Positive droplet populations were separated from negative droplets and quantified automatically as copies/μl.

### Statistical analysis

The absolute copy numbers of three mRNAs were normalized by microgram of total RNA amount from urine sample. Data were then log_10_-transformed to reduce the deviation from normality in the two PCR systems prior to statistical analysis.

Statistical analyses were conducted using the Kruskall-Wallis and Mann Whitney tests for non-parametric data using SPSS statistical software (version 20; SPSS Inc., Chicago, IL, USA). Binary logistic regression and receiver operating characteristic (ROC) curve analysis were also performed with SPSS statistical software. A *p-*value less than 0.05 was considered statistically significant.

## Results

### Clinical characteristics and samples

There was no significant difference in the mean age of patients among the ACR, AMR and stable groups (50.5 ± 11.0, 47.2 ± 11.1, and 47.2 ± 9.2, respectively, *p* = 0.358). In addition, no significant differences were observed between the groups regarding the time since kidney transplant, but HLA mismatch was statistically significant between the stable and AMR groups. At the time of graft biopsy, serum creatinine levels and eGFR in both the ACR and AMR groups were significantly higher than in the control group (*p* < 0.001). Clinical characteristics of the study population are summarized in Table 1.

**Table 1:** Clinical characteristics of kidney allograft recipients.

Clinical characteristics	Stable graft function	Acute cellular rejection	Acute antibody-mediated rejection	Stable vs ACR‡ (by student T-test)	ACR vs AMR‡	Stable vs AMR‡	*p*-value (by ANOVA) †
Kidney allograft patients, *N*	44	29	15				
Urine samples, *N*	44	31	15				
Male, %	17 (38.6)	21 (67.7)	11 (73.3)	0.005	0.948	0.020	0.006
Age (yr)	50.5±11.01	47.2±11.1	47.2±9.2	0.210	0.998	0.307	0.358
Time since KT* (days)	3652.5 (37.3–4960.3)	235.0 (86.0–558.0)	498.0 (56.0–1352.0)	0.034	0.237	0.254	0.075
Serum creatinine*	1.0 (0.8–1.1)	1.8 (1.4–2.5)	2.6 (1.7–3.8)	<0.001	0.073	<0.001	<0.001
eGFR by MDRD	76.3±18.3	47.3±9.2	30.9±17.0	<0.001	0.128	<0.001	<0.001
HLA mismatch*	3 (1-4-5)	4 (2–5)	4 (3–5)	0.136	0.389	0.030	0.069

* Data are expressed as the medians and interquartile ranges (IQR) due to non-normal distributions.

^†^ For non-normally distributed variables, data were analyzed using the Kruskal-Wallis test.

^‡^ For non-normally distributed variables, data were analyzed using the Mann Whitney U test.

For categorical variables, data were analyzed using Pearson’s chi-square test.

**Fig 1:**
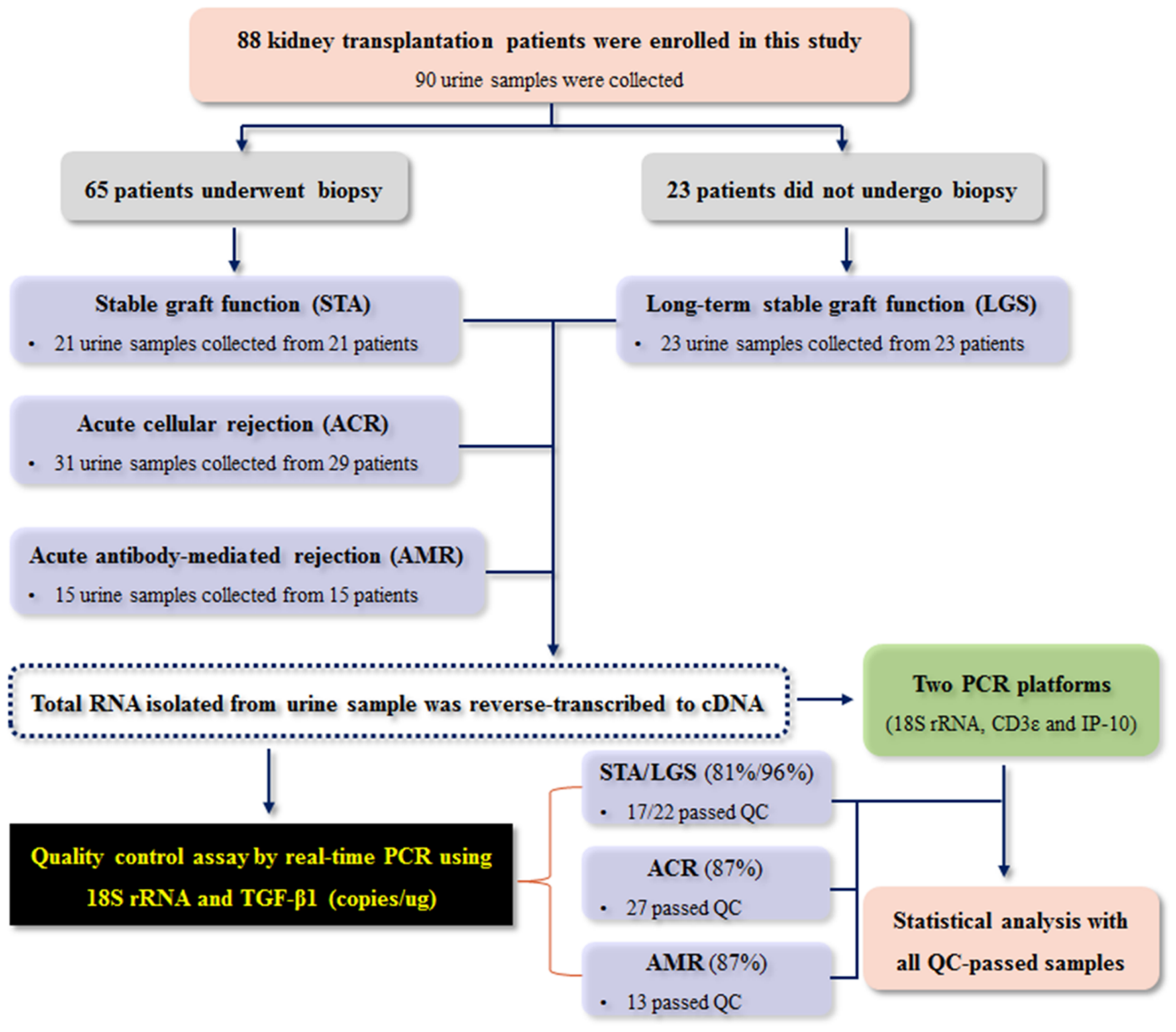
Workflow of patients, urine samples, and experimental design. For validation of urinary cell mRNA, 90 urine samples were collected from 88 kidney transplant patients. Two PCR platforms were used to process the 90 samples, and 79 of the samples passed the quality control (QC) thresholds of TGF-β1 and 18S rRNA copies/μg total RNA; 11 did not pass. Data were statistically analyzed with the QC-passed samples.

To validate mRNA levels in urinary cells, 90 urine samples were collected from 88 kidney transplant patients. Of these 90 samples, 67 samples were obtained at the time of graft biopsy, and the remaining 23 samples were obtained from patients who did not undergo biopsy because they exhibited long-term good survival (LGS). The samples were divided into three groups: stable graft function (STA, n = 44), including LGS; acute cellular rejection (ACR, n = 31); and acute antibody-mediated rejection (AMR, n = 15). We performed this experiment to validate expression levels of mRNA isolated from urinary cells (Fig 1). Copy numbers of 18S rRNA and TGF-β1 per microgram of total RNA assessed by quantitative real-time PCR were used for quality control; out of the 90 urine samples, 79 samples (39 STA, 27 ACR, and 13 AMR) passed quality control and 11 did not. All results were presented with data of the QC-passed samples.

### Validation of urinary mRNA levels to predict acute rejection

**Fig 2:**
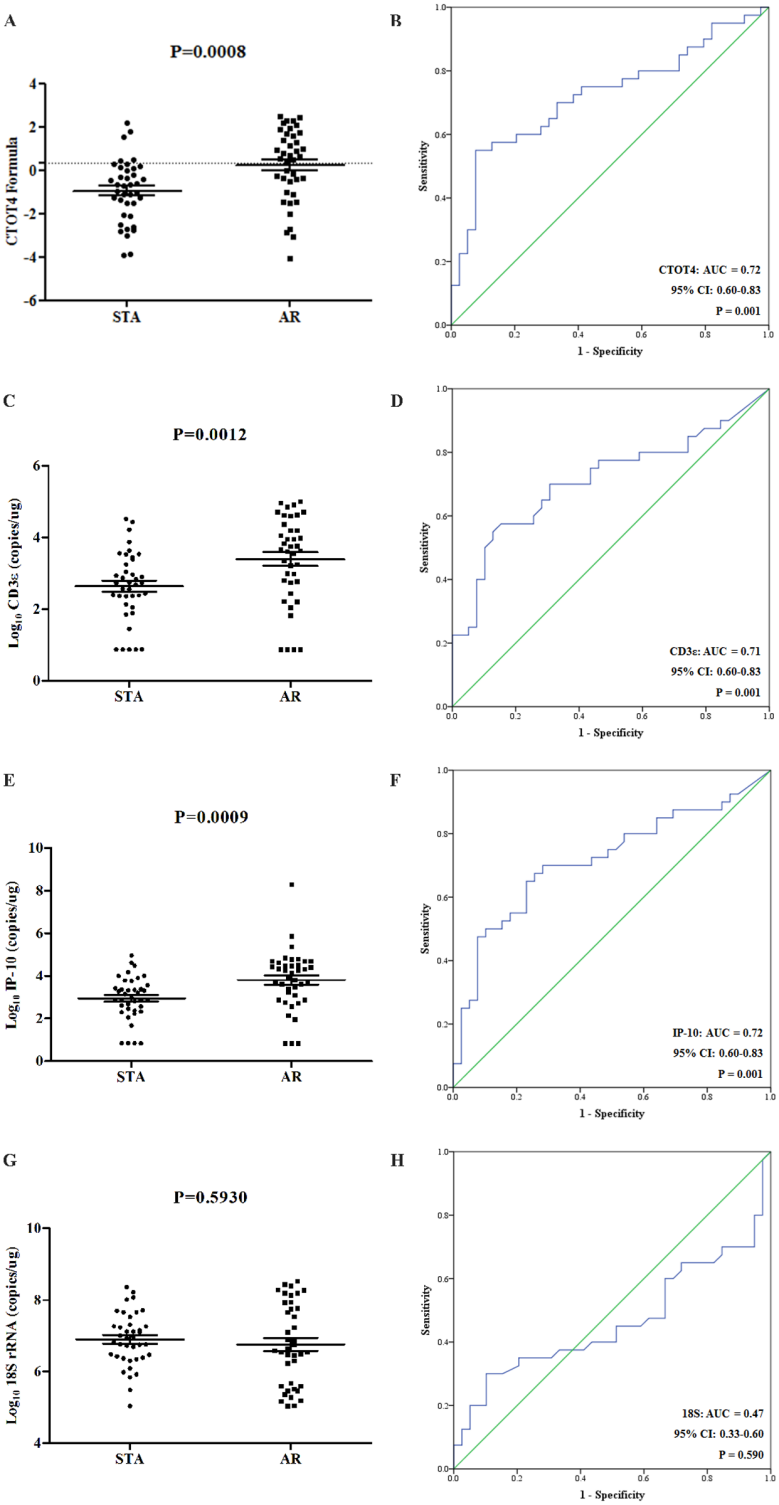
The mRNA levels of the CTOT4 formula and CD3ε, IP-10, and 18S rRNA in qPCR. (A) The mRNA levels of the CTOT4 formula, (C) CD3ε, (E) IP-10, and (G) 18S rRNA between two groups were analyzed by qPCR, respectively. (B, D, F, H) The results corresponding ROC curve analyses for the CTOT4 formula and three genes, respectively.

We tried to validate the molecular signature of the CTOT-4 study in Korean kidney transplant recipients by quantitative real-time PCR with standard curve of each target for the absolute quantification. Compared with stable group using the CTOT-4 formula (F = -6.1487 + 0.8534 log_10_ (CD3ε) + 0.6376 log_10_ (IP-10) + 0.1554 log_10_ (18S)), the signature increased in AR group (p = 0.0008), with AUC of 0.72 (95% confidence interval [CI], 0.60–0.83; p = 0.001) (Fig 2A and 2B). Our result was similar to that of previously reported results.

We also assessed each of three urinary mRNAs (18S rRNA, CD3ε and IP-10 mRNA) reported in the CTOT-4 study. The values in the PCR assay were the log_10_ transformations of copy number per microgram of total RNA for three genes. Compared with stable group, the values of CD3ε (p = 0.0012) and IP-10 (p = 0.0009) were significantly elevated in AR group, with AUC of 0.71 and 0.72, respectively (95% CI, 0.60–0.83; p = 0.001) (Fig 2C–2F). However, the value of 18S rRNA was not different between patient groups (AUC = 0.47, p = 0.593) in our study (Fig 2G and 2H) and not consistent with the result of previously published study.

### Validation of the urinary mRNAs using droplet digital PCR

**Fig 3:**
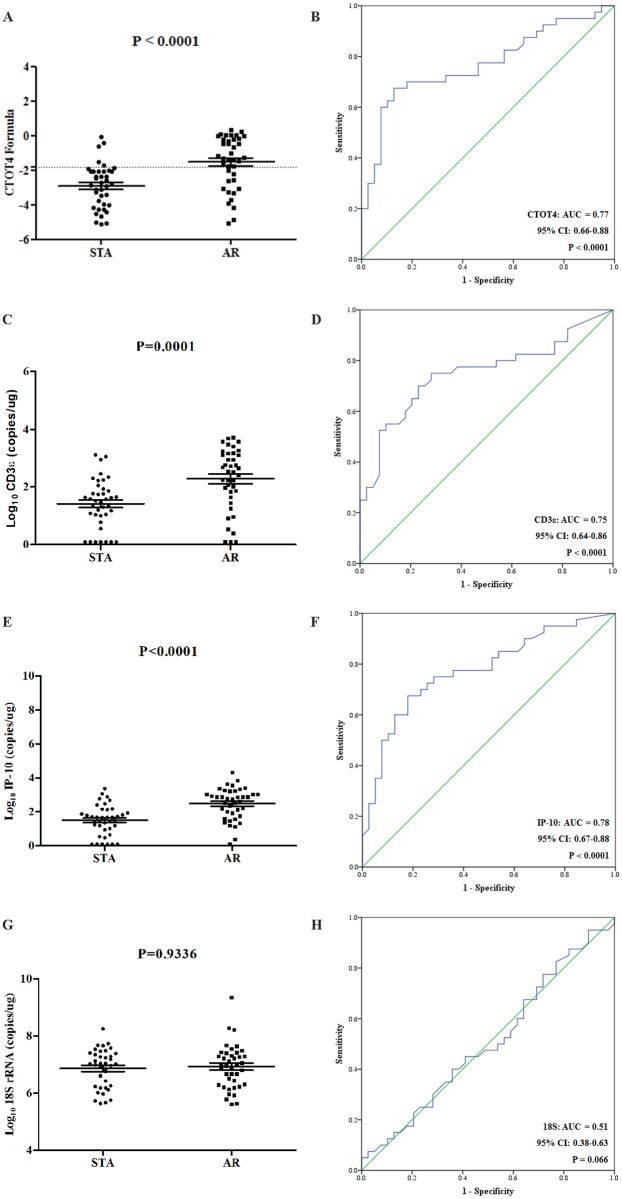
The mRNA levels of the CTOT4 formula and the mRNAs in ddPCR. (A) The CTOT4 formula, (C) CD3ε, (E) IP-10, and (G) 18S rRNA represented the absolute mRNA levels between two groups analyzed by ddPCR, respectively. (B, D, F, H) The results corresponding ROC curve analyses for the CTOT4 formula and three genes, respectively.

We used the next-generation droplet digital PCR (ddPCR) with many potential advantages including absolute quantification, sensitivity, accuracy, and reproducibility. Compared with stable group using the CTOT-4 formula, the signature increased in AR group (p < 0.0001), with AUC of 0.77 (95% CI, 0.66–0.88; p < 0.0001) (Fig 3A and 3B).

We then analyzed each of three mRNAs. Compared with stable group, the values of CD3ε (p = 0.0001) and IP-10 (p < 0.0001) were significantly elevated in AR group, with AUC of 0.75 (95% CI, 0.64–0.86; p < 0.0001) and 0.78 (95% CI, 0.67–0.88; p < 0.0001), respectively (Fig 3C–3F). As expected with the result of real-time PCR, 18S rRNA was not different between patient groups (AUC = 0.51, p = 0.9336) (Fig 3G and 3H).

Taken together, these results showed that the signature reported in CTOT-4 study was a good diagnostic marker for AR and the validation by the ddPCR analysis was comparable to the result from real-time PCR analysis. The ddPCR, which doesn't need standard curve, may be considered as a useful tool for clinical application. In addition, the 18S rRNA determined in two PCR platforms was unable to discriminate between groups in our study. Consequently, we thought that 18S as a reference gene may be used for the normalization of urinary mRNAs.

### Strategies to analyze gene expression data in PCR systems

**Fig 4:**
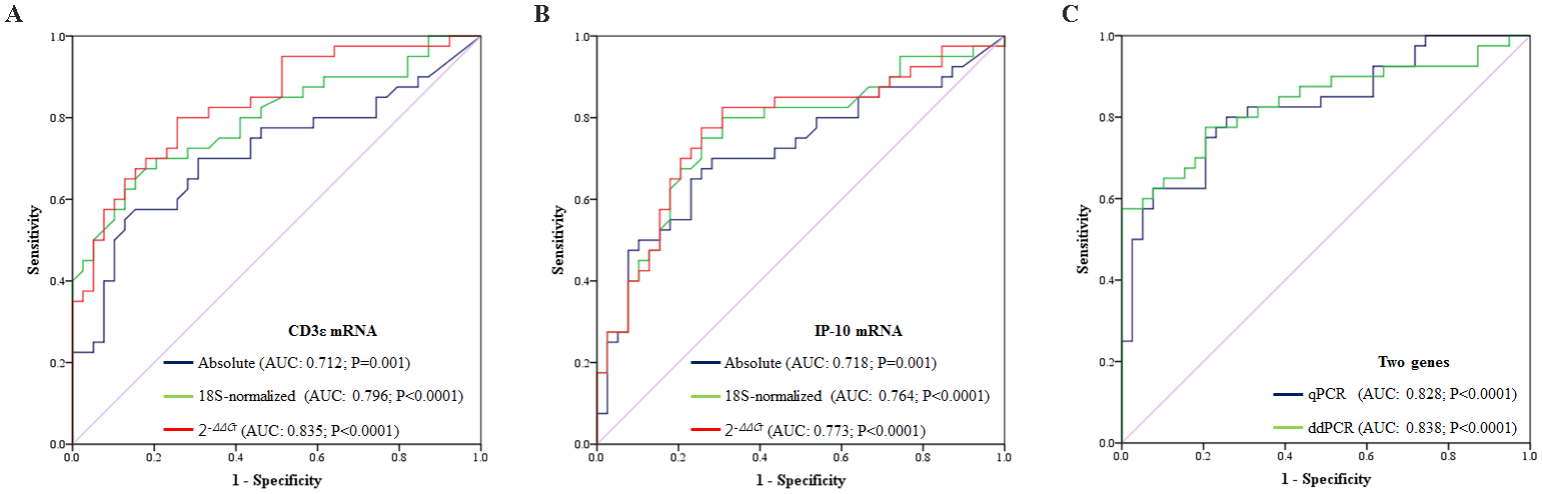
Comparison of several methods for mining data and two PCR platforms. (A-B) **T**he area under the curve (AUC) values for urinary CD3ε and IP-10 mRNA levels normalized by the total RNA amount (absolute), absolute copy number of 18S rRNA, or the 2^-*ΔΔC*т^ method in qPCR platform. (C) ROC curves for two-gene set (CD3ε and IP-10) normalized by absolute copy number of 18S rRNA in qPCR and ddPCR platforms.

The values of CD3ε and IP-10 mRNAs per microgram of total RNA well discriminated between AR and stable group, but not 18S in our result. We thus normalized the CD3ε and IP-10 mRNAs with copy number of 18S and with *C*т value of 18S as the most widely used reference gene and then compared the discrimination capacities of two genes using the absolute, 18S-normalized, and 2^-*ΔΔC*т^ method in qPCR analysis. The AUC values of 18S normalization and 2^-*ΔΔC*т^ method for CD3ε mRNA were 0.80 (95% CI, 0.70–0.90; p < 0.0001) and 0.84 (95% CI, 0.75–0.92; p < 0.0001), respectively (Fig 4A), and those of 18S normalization and 2^-*ΔΔC*т^ method for the IP-10 were 0.76 (95% CI, 0.66–0.87; p < 0.0001) and 0.77 (95% CI, 0.67–0.88; p < 0.0001), respectively (Fig 4B). The results by 18S normalization were better than those of the absolute copy number, and although there was no statistical significance, the AUC value by the 2^-*ΔΔC*т^ method was numerically high. Furthermore, to compare the results of qPCR and ddPCR for diagnosis of AR, we performed binary logistic regression with the 18S-normalized CD3ε and IP-10 mRNAs. The AUC value of the two-gene signature in ddPCR was numerically higher than those in qPCR (Fig 4C).

## Discussion

Acute rejection is an important obstacle for long-term graft survival in transplant recipients. Although graft biopsy has been used to monitor certain kidney conditions, it is inherently invasive and problematic, as inter-observer variability and complications often occur [16, 17]. Because of these limitations of the renal biopsy, non-invasive diagnostic tools are necessary to manage early graft rejection and to improve graft survival. Currently, absolute quantification of the urinary cell mRNAs for CD3ε, IP-10, and 18S rRNA as non-invasive method to diagnose acute rejection was developed by Suthanthiran group [2], and the PCR method is quite promising to be translated into clinical practice. The assessment of urinary mRNA with information on kidney allograft status is good way to non-invasively monitor kidney allograft damage, while urinary mRNA has low stability in general. Recently, Galichon et al suggested the issue of normalization to ensure the reproducibility and to suppress the effect of RNA degradation in urine samples because of the characteristic of urinary mRNA [4].

We agree with this normalization issue because of the aforementioned limitation of urinary mRNA. In spite of the limitation, if the PCR-based protocol is consistent and the diagnostic biomarkers could be validated by independent groups, quantification of urinary mRNA can certainly be helpful in the non-invasive diagnosis for kidney transplantation recipients. Our study validated diagnostic performance of biomarker for AR using three genes reported by Suthanthiran group in qPCR and ddPCR platforms and focused on easy and simple analyzed method of the PCR-based data in urinary mRNA expression for clinical application.

Quantitative real-time PCR (qPCR) system has been favorably and conveniently used by many researchers, and absolute and relative quantification are commonly used to analyze data. While the qPCR method has been well established, there are some remaining limitations, such as low sensitivity and the requirements of a reference sample, an endogenous control and a standard curve for absolute quantification. Recently, digital PCR has become widely used for research and clinical applications because of advantages such as high sensitivity, accuracy and reproducibility, and in contrast with qPCR, absolute quantification of nucleic acids without standard curves [18–20]. Digital PCR can be used to detect mutations, analyze copy number variations, and quantify specific nucleic acid species [21]; it has proven useful for the analysis of cancer genetic variations[13], heterogeneous methylation [22], fetal screening [23], biomarker analysis [24], viral detection, and mitochondrial DNA alterations in Alzheimer disease [25] and others [26]. Moreover, the advanced ddPCR (Bio-Rad QX200) system has shown better reliability at low target concentrations and a greater tolerance for inhibitors [27]. Thus, we compared two quantitative PCR platforms in validation of known three genes in urinary cells of Korean kidney transplant recipients.

We also slightly modified a previously used method, which included a pre-amplification step prior to quantification [2]. In the modified PCR method, total RNA from urinary cells was eluted with 30 μl RNase-free water, standard curves prepared with DNA fragments of each target were used for absolute quantification, and mRNAs did not perform pre-amplification step for fast, easy, and simple quantification. The urine samples passed quality control if the 18S rRNA copy number was ≥ 5x10^5^ per microgram of total RNA and the TGF-β1 copy number was ≥ 100 per microgram of RNA. By these criteria, 88% of urine samples passed quality control and this rate was comparable with that (83%) of the original study. We thus validated the CTOT-4 formula for quantification of urinary mRNAs in Korean kidney transplant recipients, who have different genetic and demographic features from American kidney transplant recipients and compared diagnostic performance for AR using two PCR systems. qPCR results were similar to those of previously reported results, and the results by ddPCR was comparable, but a little bit higher AUC results than those by qPCR.

In our study, because 18S rRNA was not significantly different between all patient groups, we used 18S rRNA as reference gene and the copy numbers of CD3ε and IP-10 mRNA were normalized by both 18S rRNA copies (x10^-6^) per microgram of total RNA and microgram of total RNA. 18S normalization showed higher AUC results than those of total RNA normalization for both CD3ε and IP-10. These results were not consistent with previous results, which showed 18S expression was slightly, but significantly increased in AR patients as compared with stable patients. This discrepancy could be explained by the differences in sample size, heterogeneity in stable patients (including long-term good survivals in this study) and possible variation in pre-amplification step in previous studies. We also we compared the relative 2^-*ΔΔC*т^ method with the absolute quantification method in quantitative real-time PCR analysis. The relative 2^-*ΔΔC*т^ method is generally used in analysis of PCR data [10, 11, 14]. 18S as reference gene and commercially universal reference RNA to reduce batch effect were used for the 2^-*ΔΔC*т^ method. The results of CD3ε and IP-10 mRNA normalized by 18S had better association with AR than those normalized by the absolute copy number of 18S, and the urinary mRNAs by the 2^-ΔΔCт^ method yielded numerically the best AUC for the diagnosis of AR. We then performed binary logistic regression with the relative quantification using 2^-*ΔΔC*т^ method for CD3ε and IP-10 mRNAs to compare diagnostic performances for AR of both qPCR and ddPCR. The AUC values of the relative expression to 18S for two-gene signature were improved in both PCR system (0.828 and 0.838, respectively).

In conclusion, our study validated the usefulness of CTOT-4 formula in Asian recipients. We have demonstrated that a pre-amplification step might not be necessary for the quantification of urine mRNA for limited number of target genes. Except for the 18S rRNA data, our results were consistent with those of the previous study [2]: the two genes normalized by 18S clearly distinguished between AR and STA in Korean kidney transplant recipients. Also, if the chosen reference gene is stable between sample groups according to experimental condition and samples, the relative as well as absolute quantification in urine samples can be useful to monitor kidney allograft rejection in real-time PCR analysis. Furthermore, we first applied the ddPCR system with the benefits, such as absolute quantification, accuracy, and reproducibility, to non-invasively monitor for AR after kidney transplantation. Because a standard curve in ddPCR system is dispensable for absolute quantification, the system can be useful for determining gene expression levels in urinary cells. However, further validation of this gene signature’s ability to distinguish patients with AR from those with other graft conditions will be required in a larger cohort prior to clinical trials.

## Supporting information

S1 Table Baseline clinical characteristics of patients with renal allograft recipients. (DOCX)

S2 Table Banff scores and percent of renal allograft recipients with histology scores >0 on biopsy. (DOCX)

S3 Table Oligonucleotide primers and probes used for the quantification of RNAs. (DOCX)

S4 Table Total quantity and purity of RNA extracted from urine cells. (DOCX)

S5 Table Absolute levels and log_10_ 18S rRNA-normalized levels of mRNA in qPCR. (DOCX)

S6 Table Absolute levels and log_10_ 18S rRNA-normalized levels of mRNA in ddPCR. (DOCX)

S1 Fig The mRNA levels in 4 groups. (DOCX)
