# Supplementary material for: Both absolute and relative quantification of urinary mRNA are useful for non-invasive diagnosis of acute kidney allograft rejection
Source: PLoS One. 2017 Jun 27;12(6):e0180045. doi: 10.1371/journal.pone.0180045 (PMC5487057; doi:10.1371/journal.pone.0180045)
Supplement: S1 Table — (DOCX) [file pone.0180045.s001.docx]

**S1 Table. Baseline clinical characteristics of patients with renal allograft recipients.**

|  | **STA/LGS (n=44)** | **ACR (n=31)** | **AMR (n=15)** | ***p^†^*** |
| --- | --- | --- | --- | --- |
| **Donor information** |  |  |  |  |
| Age (years) | 39.1±13.6 | 50.2±10.2 | 43.4±13.1 | 0.002 |
| Deceased donor (n, %) | 18 (40.9) | 18 (58.1) | 7 (46.7) | 0.144 |
| ABO incompatible (n, %) | 4 (9.1) | 4 (12.9) | 5 (33.3) | 0.057 |
| **Immunosuppressive regimen** |  |  |  |  |
| Steroid (n, %) | 29 (67.4) | 28 (90.3) | 15 (100) | 0.006 |
| Calcineurin inhibitor (n, %) | 40 (90.9) | 30 (96.8) | 14 (93.3) | 0.269 |
| Mycophenolate mofetil (n, %) | 27 (62.8) | 22 (71.0) | 13 (86.7) | 0.219 |
| mTOR inhibitor (n, %) | 5 (11.6) | 0 (0) | 1 (6.7) | 0.232 |
| **Banff classification of rejection** |  | 4-IA: 19 (61.3) | 2-I: 2 (13.3) |  |
|  |  | 4-IB: 3 (9.7) | 2-II: 12 (80.0) |  |
|  |  | 4-IIA: 7 (22.6) | 2-III: 1 (6.7) |  |
|  |  | 4-IIB:2 (6.4) |  |  |

† For non-normally distributed variables, data were analyzed using the Kruskal-Wallis test.

For categorical variables, data were analyzed using Pearson’s chi-square test.

STA, stable; LGS, long-term graft survival; ACR, acute cellular rejection; AMR, acute antibody-mediated rejection.
