# Supplementary material for: Both absolute and relative quantification of urinary mRNA are useful for non-invasive diagnosis of acute kidney allograft rejection
Source: PLoS One. 2017 Jun 27;12(6):e0180045. doi: 10.1371/journal.pone.0180045 (PMC5487057; doi:10.1371/journal.pone.0180045)
Supplement: S2 Table — (DOCX) [file pone.0180045.s002.docx]

**S2 Table. Banff scores and percent of renal allograft recipients with histology scores >0 on biopsy.**

| **Banff pathologic scores** | **STA (n=21)** | **ACR (n=31)** | **AMR (n=15)** | ***p***^†^ |
| --- | --- | --- | --- | --- |
| **Tubulitis (t)** |  |  |  |  |
| Mean t score | 0.0±0.0 | 2.3±0.7 | 0.7±1.1 | <0.001 |
| Patients with t score > 0 (n, %) | 0 (0) | 31 (100) | 6 (60.0) | 0.002 |
| **Total interstitial inflammation (ti)** |  |  |  |  |
| Mean ti score | 0.1±0.3 | 2.3±0.8 | 1.0±1.3 | <0.001 |
| Patients with ti score > 0 (n, %) | 2 (9.5) | 31 (100) | 7 (46.7) | 0.005 |
| **Intimal arteritis (v)** |  |  |  |  |
| Mean v score | 0.0±0.0 | 0.3±0.5 | 0.2±0.4 | 0.087 |
| Patients with v score > 0 (n, %) | 0 (0) | 7 (22.6) | 3 (20.0) | 0.077 |
| **Glomerulitis (g)** |  |  |  |  |
| Mean g score | 0.0±0.0 | 0.5±0.8 | 1.9±1.2 | <0.001 |
| Patients with g score > 0 (n, %) | 0 (0) | 11 (35.5) | 11 (73.3) | <0.001 |
| **Chronic tubular atrophy (ct)** |  |  |  |  |
| Mean ct score | 0.1±0.3 | 0.7±0.1 | 0.9±0.1 | 0.001 |
| Patients with ct score > 0 (n, %) | 2 (9.5) | 19 (61.3) | 10 (66.7) | <0.001 |
| **Chronic interstitial fibrosis (ci)** |  |  |  |  |
| Mean ci score | 0.1±0.3 | 0.7±0.7 | 1.0±0.9 | 0.002 |
| Patients with ci score > 0 (n, %) | 2 (9.5) | 18 (58.1) | 9 (60.0) | 0.001 |
| **Chronic vascular changes (cv)** |  |  |  |  |
| Mean cv score | 0.1±0.4 | 0.4±0.9 | 0.3±0.6 | 0.436 |
| Patients with cv score > 0 (n, %) | 1 (4.8) | 5 (16.1) | 4 (26.7) | 0.077 |
| **Transplant glomerulopathy (cg)** |  |  |  |  |
| Mean cg score | 0.0±0.0 | 0.0±0.0 | 0.2±0.8 | 0.190 |
| Patients with cg score > 0 (n, %) | 0 (0) | 0 (0) | 1 (6.7) | 0.140 |
| **Peritubular capillaritis (ptc)** |  |  |  |  |
| Mean ptc score | 0.0±0.0 | 0.3±0.7 | 2.3±0.8 | <0.001 |
| Patients with ptc score > 0 (n, %) | 0 (0) | 4 (12.9) | 14 (93.3) | <0.001 |

Data are expressed as mean ± standard deviation.

† For non-normally distributed variables, data were analyzed using the Kruskal-Wallis test.

For categorical variables, data were analyzed using Pearson’s chi-square test.
