# Supplementary material for: Both absolute and relative quantification of urinary mRNA are useful for non-invasive diagnosis of acute kidney allograft rejection
Source: PLoS One. 2017 Jun 27;12(6):e0180045. doi: 10.1371/journal.pone.0180045 (PMC5487057; doi:10.1371/journal.pone.0180045)
Supplement: S3 Table — (DOCX) [file pone.0180045.s003.docx]

**S3 Table: Oligonucleotide primers and probes used for the quantification of RNAs.**

| **Gene** | **Sequence** | **Location** |
| --- | --- | --- |
| IP-10 | Sens: 5' TGTCCACGTGTTGAGATCATTG 3' | 235-256 |
|  | Antisense: 5' GGCCTTCGATTCTGGATTCA 3' | 309-290 |
|  | Probe: 5’ FAM TACAATGAAAAAGAAGGGTGAGAA MGB 3’ | 258-281 |
| CD3ε | Sense: 5' AAGAAATGGGTGGTATTACACAGACA 3' | 131-156 |
|  | Antisense: 5' TGCCATAGTATTTCAGATCCAGGAT 3' | 233-209 |
|  | Probe: 5' FAM CCATCTCTGGAACCACAGTAATATTGACATGCCTAMRA 3' | 170-202 |
| 18S rRNA | TaqMan® Gene Expression Assays Hs99999901_s1 (Life Technologies) |  |
| TGF-β1 | TaqMan® Gene Expression Assays Hs00998133_m1 (Life Technologies) |  |
