# Supplementary material for: Both absolute and relative quantification of urinary mRNA are useful for non-invasive diagnosis of acute kidney allograft rejection
Source: PLoS One. 2017 Jun 27;12(6):e0180045. doi: 10.1371/journal.pone.0180045 (PMC5487057; doi:10.1371/journal.pone.0180045)
Supplement: S4 Table — (DOCX) [file pone.0180045.s004.docx]

**S4 Table: Total quantity and purity of RNA extracted from urine cells.**

| RNA Quantity | | | | |
| --- | --- | --- | --- | --- |
| Total RNA Quantity (ug) | Total samples (N=90) | QC passed^†^ (N=79) | QC Failed (N=11) | P Value^‡^  QC passed vs. QC failed |
| Median | 0.33 | 0.318 | 0.6465 | 0.1566 |
| 25% Percentile | 0.1541 | 0.153 | 0.159 |  |
| 75% Percentile | 0.6488 | 0.582 | 4.331 |  |
| RNA Purity | | | | |
| RNA purity (A260/280 ratio) | Total samples (N=90) | QC passed ^†^ (N=79) | QC Failed (N=11) | P Value^‡^  QC passed vs. QC failed |
| Median | 1.925 | 1.93 | 1.68 | 0.0325 |
| 25% Percentile | 1.81 | 1.83 | 0.5 |  |
| 75% Percentile | 2.045 | 2.06 | 2 |  |

The quantity (absorbance at 260nm) and purity (ratio of the absorbance at 260 and 280nm) of the RNA were measured using the NanoDrop® ND-2000 UV-V is spectrophotometer (Thermo Scientific).

† A RNA sample was classified as quality control passed if the 18S rRNA copy number was greater than or equal to 5 x 10^5^ per microgram of total RNA and TGF-**β**1 mRNA copy number was greater than or equal to 100 copies per microgram of RNA.

‡ Two-tailed P value is based on the Mann-Whitney test.
