# Supplementary material for: Both absolute and relative quantification of urinary mRNA are useful for non-invasive diagnosis of acute kidney allograft rejection
Source: PLoS One. 2017 Jun 27;12(6):e0180045. doi: 10.1371/journal.pone.0180045 (PMC5487057; doi:10.1371/journal.pone.0180045)
Supplement: S5 Table — (DOCX) [file pone.0180045.s005.docx]

**S5 Table: Absolute levels and log_10_ 18S rRNA-normalized levels of mRNA in qPCR.**

| **Absolute levels of mRNA in qPCR.** | | | | | | | | |
| --- | --- | --- | --- | --- | --- | --- | --- | --- |
| Type of mRNA | STA/LGS (N=39) | AR (N=40) | P Value^†^ (STA/LGS vs AR) | ACR (N=27) | AMR (N=13) | P Value^†^  (STA/LGS vs ACR) | P Value^†^  (STA/LGS vs AMR) | P Value^†^  (ACR vs AMR) |
| CD3ε | 533  (135, 2460) | 4450 (562, 21650) | 0.0012 | 4060  (555, 15800) | 8860  (1192, 41900) | 0.0072 | 0.0095 | 0.4525 |
| IP-10 | 1030  (232, 3610) | 11075 (883, 40750) | 0.0008 | 4750 (747, 29200) | 20900 (2775, 153600) | 0.0078 | 0.004 | 0.1528 |
| 18S rRNA | 20  (8, 90) | 13 (3, 146) | 0.4157 | 12 (2, 128) | 54 (4, 374) | 0.1459 | 0.554 | 0.0832 |
| TGF-β1 | 86000  (33000, 300000) | 170000 (37750, 507500) | 0.1713 | 160000 (26000, 520000) | 290000 (83000, 785000) | 0.4262 | 0.0992 | 0.3405 |
| **Log_10_ 18S rRNA-normalized levels of mRNA in qPCR.** | | | | | | | | |
| Type of mRNA | STA/LGS (N=39) | AR (N=40) | P Value^†^ (STA/LGS vs AR) | ACR (N=27) | AMR (N=13) | P Value^†^  (STA/LGS vs ACR) | P Value^†^  (STA/LGS vs AMR) | P Value^†^  (ACR vs AMR) |
| CD3ε | 1.280  (0.763, 1.690) | 2.255  (1.453, 2.783) | < 0.0001 | 2.290  (1.920, 2.800) | 1.830  (1.235, 2.670) | < 0.0001 | 0.0179 | 0.3053 |
| IP-10 | 1.530  (0.956, 2.160) | 2.540  (2.065, 3.230) | < 0.0001 | 2.540  (2.140, 3.330) | 2.540  (1.345, 3.015) | < 0.0001 | 0.0185 | 0.6649 |

Levels of mRNA were measured by real-time quantitative PCR assays using standard curve. Median absolute copy number per microgram of total RNA (lower, upper quartiles) of each mRNA measure without log_10_-transformation and normalized by 18s rRNA copy number (x10^-6^) per microgram of total RNA without log_10_-transformation are shown in QC-passed urine samples.

† Two-tailed P value is based on the Mann-Whitney test.
