# Supplementary material for: Both absolute and relative quantification of urinary mRNA are useful for non-invasive diagnosis of acute kidney allograft rejection
Source: PLoS One. 2017 Jun 27;12(6):e0180045. doi: 10.1371/journal.pone.0180045 (PMC5487057; doi:10.1371/journal.pone.0180045)
Supplement: S6 Table — (DOCX) [file pone.0180045.s006.docx]

**S6 Table: Absolute levels and log_10_ 18S rRNA-normalized levels of mRNA in ddPCR.**

| **Absolute levels of mRNA in ddPCR.** | | | | | | | | |
| --- | --- | --- | --- | --- | --- | --- | --- | --- |
| Type of mRNA | STA/LGS (N=39) | AR (N=40) | P Value^†^  STA/LGS vs AR | ACR (N=27) | AMR (N=13) | P Value^†^  (STA/LGS vs ACR) | P Value^†^  (STA/LGS vs AMR) | P Value^†^  (ACR vs AMR) |
| CD3ε | 33 (10, 86) | 333 (51, 1388) | 0.0001 | 250 (45, 1256) | 444 (89, 1646) | 0.0007 | 0.0057 | 0.6234 |
| IP-10 | 46 (9, 133) | 590 (64, 1534) | < 0.0001 | 564 (83, 1131) | 733 (41, 2246) | < 0.0001 | 0.0068 | 0.8624 |
| 18S rRNA | 10 (2, 24) | 9 (2, 24) | 0.9492 | 7 (2, 19) | 19 (3, 40) | 0.5705 | 0.2859 | 0.1841 |
| **Log_10_ 18S rRNA-normalized levels of mRNA in ddPCR.** | | | | | | | | |
| Type of mRNA | STA/LGS (N=39) | AR (N=40) | P Value^†^  STA/LGS vs AR | ACR (N=27) | AMR (N=13) | P Value^†^  (STA/LGS vs ACR) | P Value^†^  (STA/LGS vs AMR) | P Value^†^  (ACR vs AMR) |
| CD3ε | 0.464 (0.126, 0.978) | 1.495 (0.890, 1.993) | < 0.0001 | 1.730 (0.846, 2.090) | 1.240 (0.817, 1.845) | 0.0001 | 0.0058 | 0.3191 |
| IP-10 | 0.490 (0.126, 1.150) | 1.650 (1.200, 2.098) | < 0.0001 | 1.640 (1.200, 2.120) | 1.660 (0.823, 1.990) | < 0.0001 | 0.002 | 0.5158 |

Levels of mRNA were measured by droplet digital PCR assays without standard curve. Median absolute copy number per microgram of total RNA (lower, upper quartiles) of each mRNA measure without log_10_-transformation and normalized by 18s rRNA copy number (x10^-6^) per microgram of total RNA without log_10_-transformation are shown in QC-passed urine samples.

† Two-tailed P value is based on the Mann-Whitney test.
