# Supplementary figures and images for: Both absolute and relative quantification of urinary mRNA are useful for non-invasive diagnosis of acute kidney allograft rejection
Source: PLoS One. 2017 Jun 27;12(6):e0180045. doi: 10.1371/journal.pone.0180045 (PMC5487057; doi:10.1371/journal.pone.0180045)

**S1 Figure: The mRNA levels in 4 groups.**


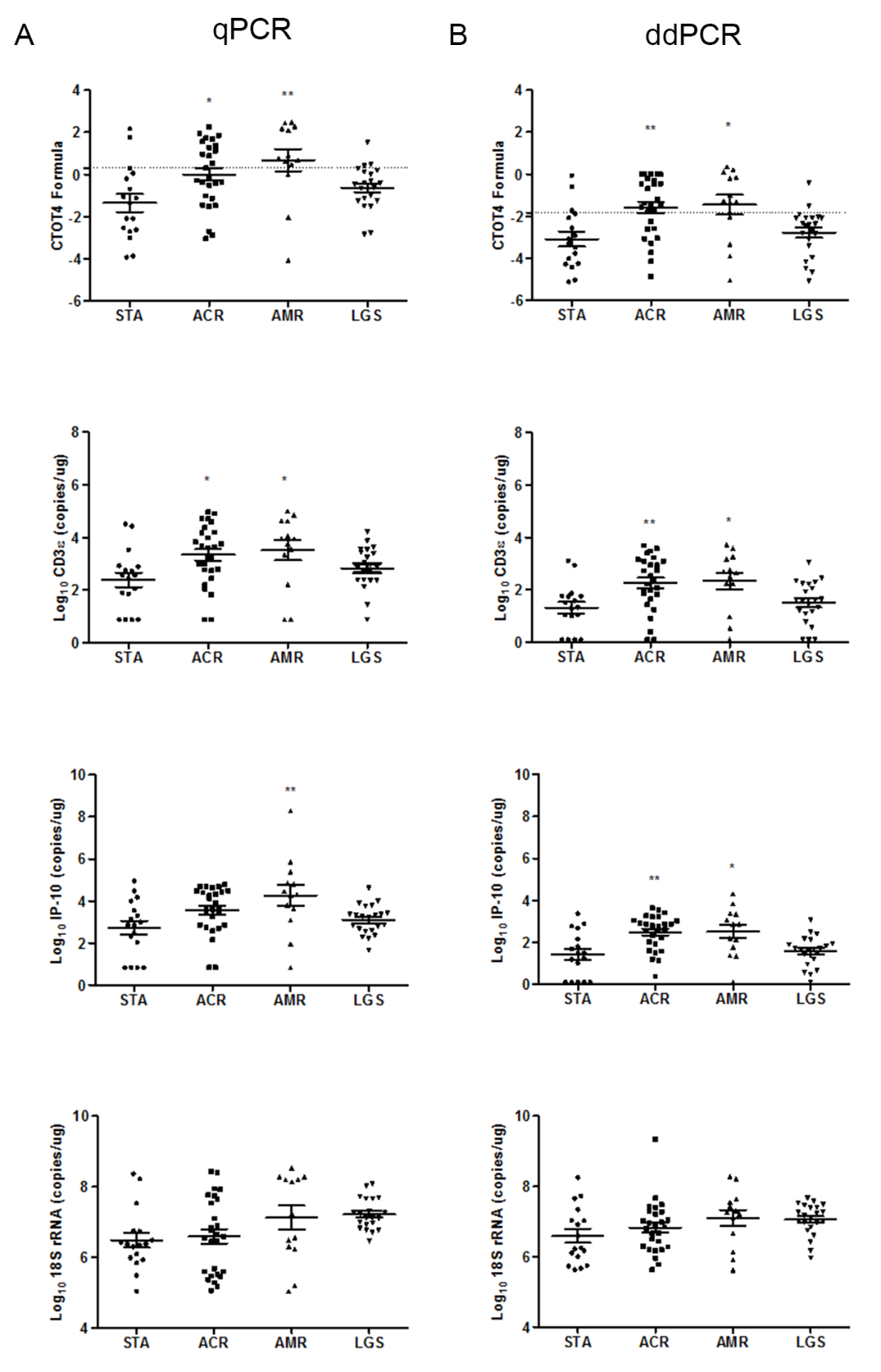

Supplement: S1 Fig — (DOCX) [file pone.0180045.s007.docx]
